# The complete mitochondrial genome of *Boulenophrys sanmingensis* (Anura: Megophryidae)

**DOI:** 10.1080/23802359.2026.2663604

**Published:** 2026-04-29

**Authors:** Zi-Yi Zheng, Xiao-Hua Guo, Guo-Hua Ding, Jing Chen

**Affiliations:** a School of Life Sciences, Zhejiang Chinese Medical University, Hangzhou, China; b Ecological Forestry Development Center, Lishui, China; c College of Agriculture and Biotechnology, Lishui University, Lishui, China

**Keywords:** Mitogenome, phylogenetics, *Boulenophrys*, East China

## Abstract

The complete mitochondrial genome (mitogenome) of *Boulenophrys sanmingensis* (Anura: Megophryidae) was sequenced and assembled using next-generation sequencing technology. The circular mitogenome is 17,414 bp in length with a GC content of 41.0%, comprising 13 protein-coding genes (PCGs), two rRNA genes, 22 tRNA genes, and a control region. One PCG (*nad6*) and eight tRNA genes are located on the light strand. Phylogenetic analysis based on 13 PCGs and two rRNA genes revealed that *B. sanmingensis* formed a well-supported clade with other *Boulenophrys* species, with *B. baishanzuensis* as its closest relative.

## 1. Introduction

*Boulenophrys sanmingensis* (Lyu and Wang 2021) is a newly described species of Asian horned toad from southeastern China, formally published in 2021, and is primarily distributed in the hilly montane areas along the border of Fujian and Jiangxi provinces (Lyu et al. 2021). The species is a small-bodied frog, characterized by the absence of vomerine teeth, a notched tongue margin, distinct tympanum, overlapping heels, wide lateral fringes and rudimentary web on toes, and a rough dorsal skin bearing an ‘X’-shaped ridge (Lyu et al. 2021). The species was originally assigned to the genus *Panophrys* (Lyu et al. 2021), but was subsequently transferred to *Boulenophrys* following ongoing revisions to the classification of Asian horned toads (Lyu et al. 2023). The genus *Boulenophrys* was first proposed by Chen et al. (2017) within a five-genus classification of the subfamily Megophryinae. Subsequent studies proposed alternative generic arrangements, including a seven-genus taxonomy (Lyu et al. 2021; Qi et al. 2021) and, most recently, a 10-genus classification (Lyu et al. 2023), which represents the currently accepted framework. *Boulenophrys* has been consistently recovered as a monophyletic clade within the subfamily Megophryinae in phylogenetic analyses (Wang et al. 2024; Wu et al. 2024). However, the generally conservative morphology of Asian horned toads makes it difficult to accurately delimit species and generic boundaries based solely on traditional morphological characters, resulting in a long-standing underestimation of diversity within this group (Chen et al. 2017; Mahony et al. 2017).

Complete mitochondrial genomes provide important molecular evidence for species identification and phylogenetic analysis, and contribute to elucidating genomic structural features and evolutionary relationships among related lineages (Xing et al. 2025). Nevertheless, as of March 2026, only eight complete mitochondrial genomes of *Boulenophrys* species are available in GenBank (accessed March 2026), despite the genus comprising 81 recognized species (Frost 2026), indicating that mitogenome data for this genus remain very limited. Further characterization of mitochondrial genomes across the genus is therefore of considerable importance for advancing our understanding of its species diversity and phylogenetic relationships.

In this study, we assembled and annotated the complete mitochondrial genome of *B. sanmingensis* using next-generation sequencing technology, characterized its genomic composition and structural features, and investigated its phylogenetic position in relation to closely related taxa, with the aim of providing baseline molecular data for systematic studies of *Boulenophrys*.

## 2. Materials and methods

### 2.1. Sample collection

**Figure 1.**
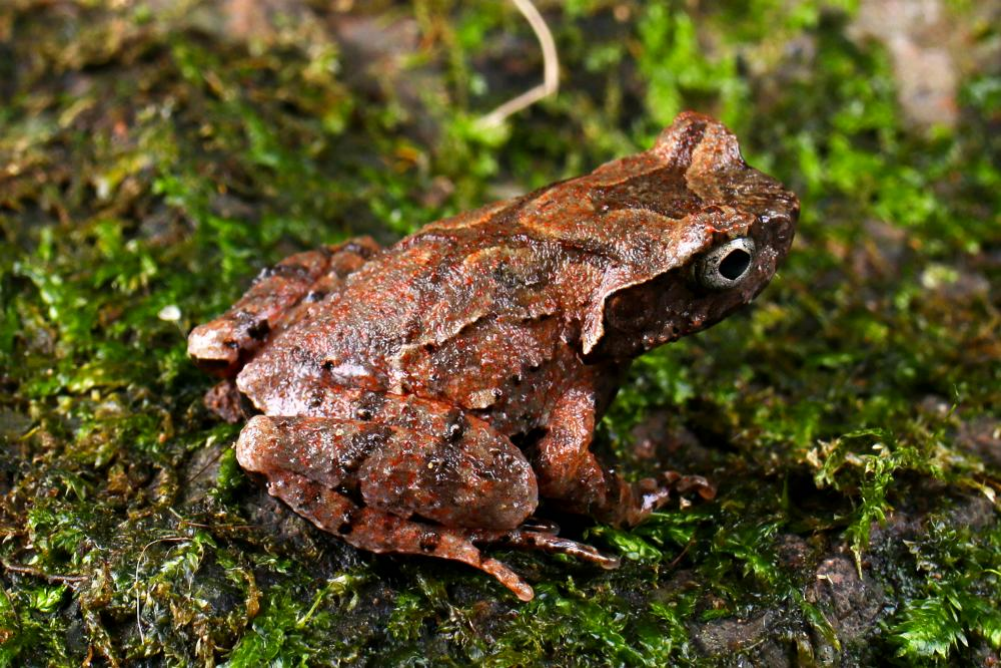
Side view of the male adult specimen of *Boulenophrys sanmingensis* used in this study. Photographed by Guo-Hua Ding.

In early July 2021, a male adult *B. sanmingensis* specimen (Figure 1) was collected from a mountain stream in Jiaoxi Village, Mingxi County, Fujian Province, China (26.52969° N, 117.20776° E; altitude: 941 m). The specimen was euthanized by immersion in MS-222 solution (10 g/L) and subsequently preserved in 70% ethanol, in accordance with AVMA Guidelines (AVMA 2020). It was deposited at the Museum of Laboratory of Amphibian Diversity Investigation at Lishui University (voucher number: LSU20210705MXWN001; contact person: Guo-Hua Ding; email: guwoding@lsu.edu.cn). The specimen was identified as *B. sanmingensis* based on morphological characters and molecular data (16S rRNA and *cox1* sequences) following Lyu et al. (2021).

### 2.2. Sequencing, genome assembly, and annotation

Muscle tissue was used to extract total genomic DNA using an EasyPure genomic DNA kit (TransGen Biotech Co., Beijing, China) following the manufacturer’s instructions. Whole genomic DNA sequencing was performed on an Illumina NovaSeq 6000 platform (Novogene Bioinformatics Technology Co. Ltd., Tianjin, China) using 150 bp paired-end reads. The mitogenome was assembled *de novo* using NOVOPlasty v3.7 (Dierckxsens et al. 2017), with a partial sequence of *Boulenophrys omeimontis* (KP728257) serving as the seed reference. Sequencing coverage was evaluated by mapping clean reads to the assembled mitogenome using Geneious Prime, yielding a mean coverage depth of 1212.9 ± 710.5× for *B. sanmingensis* (Figure S1), which confirmed the reliability and completeness of the assembly. Functional annotation of mitochondrial genes was independently performed using both MitoZ v2.4 (Meng et al. 2019) and the MITOS WebServer (Matthias et al. 2013), and the results were cross-validated for consistency. The circular mitochondrial genome map was visualized using Proksee (Grant et al. 2023). The annotated mitogenome was deposited in GenBank (accession number: PZ130025).

### 2.3. Phylogenetic analyses

The mitochondrial genome sequences of nine *Boulenophrys* species and one outgroup species were used for phylogenetic analyses. Individual genes were aligned separately using MAFFT v7.313 implemented in PhyloSuite v2 (Zhao et al. 2025). The concatenated dataset comprising 13 PCGs and two rRNA genes was used for phylogenetic inference. Maximum-likelihood (ML) analysis was performed using IQ-TREE v1.6.8, with partition-specific substitution models automatically selected by ModelFinder. The best-fit models were as follows: TIM + F + G4 for ATP6/ATP8, GTR + F + G4 for COX1, COX3, CYTB, and ND1–ND5, TPM2u + F + G4 for COX2 and ND6, and GTR + F + I + G4 for the two rRNA genes (12S rRNA and 16S rRNA). Branch support was assessed using ultrafast bootstrapping with 1000 replicates. *Atympanophrys shapingensis* (JX458090) was designated as the outgroup.

## 3. Results

### 3.1. Characteristics of *B. sanmingensis* mitogenome

**Figure 2.**
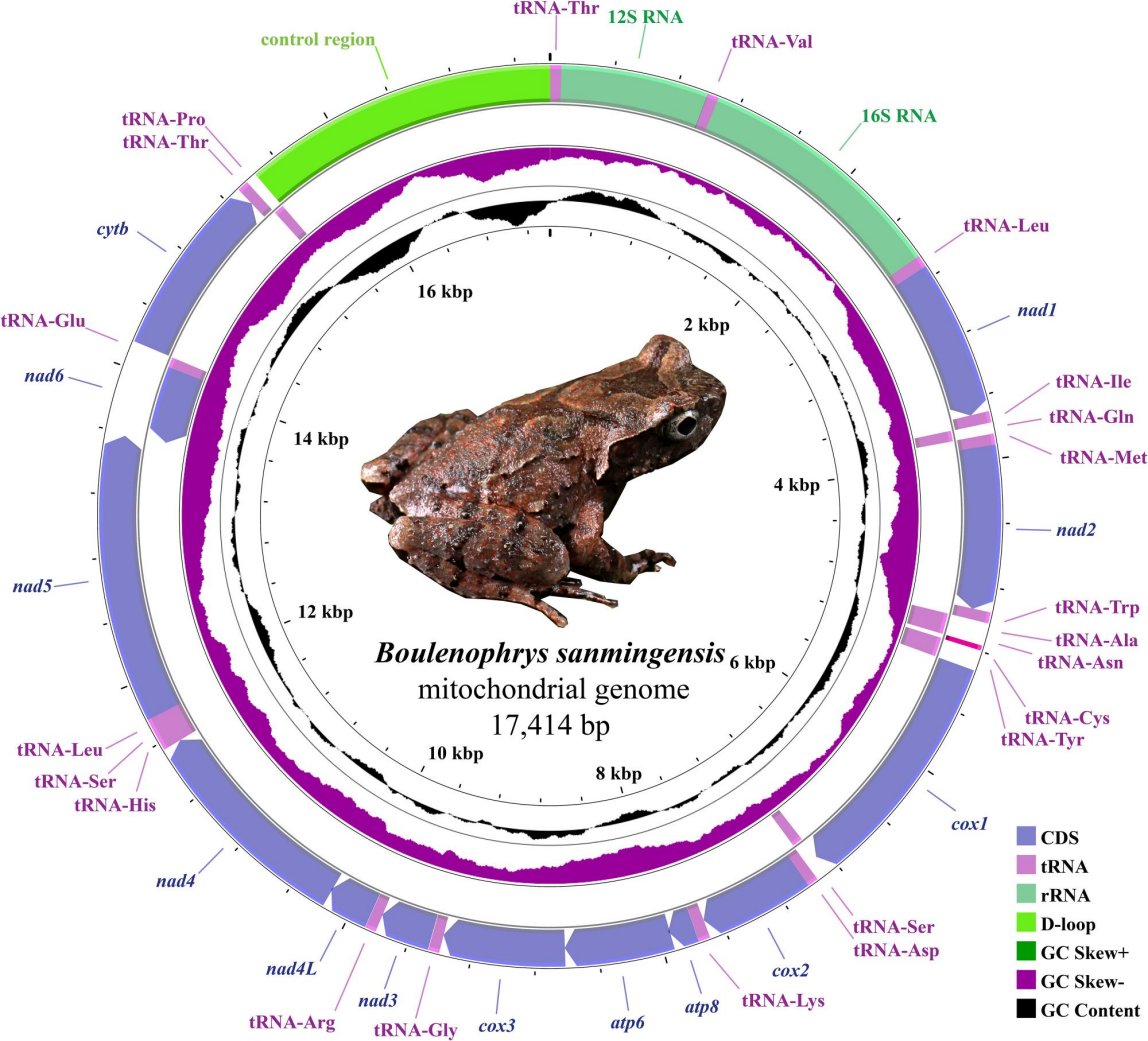
Circular map of the mitochondrial genome of *Boulenophrys sanmingensis.*

The complete mitochondrial genome of *B. sanmingensis* is 17,414 bp in length (Figure 2). The base composition is 27.4% A, 26.6% C, 14.4% G, and 31.6% T, with a GC content of 41.0%. The mitogenome consists of 13 protein-coding genes (PCGs), two rRNA genes (12S rRNA and 16S rRNA), 22 tRNA genes, and a control region (D-loop). Of the 37 genes, 28 are encoded on the heavy strand, while nine genes (nad6, tRNA-Gln, tRNA-Ala, tRNA-Asn, tRNA-Cys, tRNA-Tyr, tRNA-Ser2, tRNA-Glu, and tRNA-Pro) are located on the light strand. The *cox1* gene was initiated with GTG, the *nad2* and *nad3* genes started with ATT, and the remaining 10 PCGs started with ATG. The PCGs contained five types of stop codons: TAA (*cox1*, *atp8*, *nad4L*, and *nad5*), TAG (*nad1*, *nad2*, and *nad3*), TA (*atp6* and *cox3*), T (*cox2*, *nad4*, and *cytb*), and AGG (*nad6*).

### 3.2. Phylogenetic relationships

**Figure 3.**
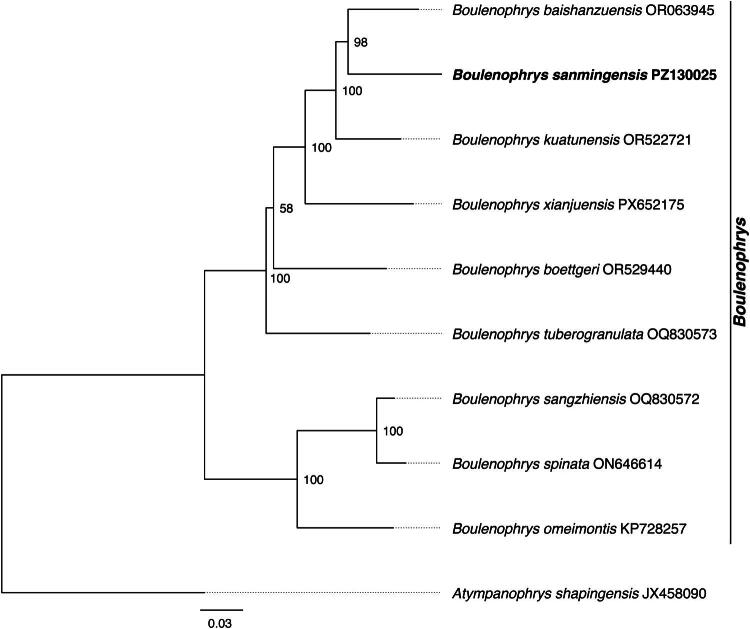
Phylogenetic tree of *Boulenophrys* based on maximum-likelihood (ML) analysis of 13 protein-coding genes and two rRNA genes. Numbers at nodes represent ultrafast bootstrap support values. The newly sequenced *B. sanmingensis* is highlighted in bold. The following sequences were used: JX458090 (Xiang et al. 2013), OQ830572 (Xiang et al. 2024), ON646614 (unpublished), OQ830573 (Xiang et al. 2024), OR063945 (Wu et al. 2024), OR522721 (Wang et al. 2024), OR529440 (Wang et al. 2024), KP728257 (Liu et al. 2016), and PX652175 (unpublished).

The ML phylogenetic tree is shown in Figure 3. The results indicated that *B. sanmingensis* was placed within a well-supported *Boulenophrys* clade, with *Boulenophrys baishanzuensis* as its closest relative (bootstrap = 98).

## 4. Discussion and conclusions

The gene arrangement and genomic composition of *B. sanmingensis* are highly consistent with those of other reported *Boulenophrys* species (Wang et al. 2024; Wu et al. 2024; Xiang et al. 2024), indicating that the mitochondrial genome structure is highly conserved within this genus. However, compared with *B. baishanzuensis*, *Boulenophrys boettgeri*, and *Boulenophrys kuatunensis*, which are co-distributed in eastern China, the control region of *B. sanmingensis* (1972 bp) is longer than that of *B. baishanzuensis* (1597 bp) and *B. boettgeri* (1146 bp), but shorter than that of *B. kuatunensis* (2471 bp), suggesting that the control region is the most variable component of the mitochondrial genome within this genus. Regarding codon usage, the stop codon of *cox1* in *B. sanmingensis* (TAA) is consistent with that of *B. baishanzuensis* and *B. kuatunensis*, but differs from that of *B. boettgeri* (AGA); the start codon of *nad2* (ATT) is shared with *B. baishanzuensis*, but differs from *B. kuatunensis* and *B. boettgeri* (ATG). These differences reflect a degree of interspecific divergence in mitochondrial genomes within *Boulenophrys*, against a background of overall structural conservation. Phylogenetic analysis supports *B. sanmingensis* as a member of a monophyletic *Boulenophrys* clade, with *B. baishanzuensis* as its closest relative, consistent with previous studies (Lyu et al. 2023; Wang et al. 2024; Wu et al. 2024), and provides new molecular evidence for further understanding of the phylogenetic relationships within this genus.

This study reports the first complete mitochondrial genome of *B. sanmingensis*, providing important baseline data for molecular systematic studies of *Boulenophrys* and contributing to the expansion of mitogenome resources for this genus.

## Supplementary Material

Author Checklist.pdf

Supplementary material.pdf

## Data Availability

The data that support the findings of this study are openly available in the GenBank of NCBI at https://www.ncbi.nlm.nih.gov under the accession number PZ130025. The associated SRA and BioSample numbers are SRR37579350 and SAMN56407955, respectively.
