# Supplementary material for: The complete mitochondrial genome of Boulenophrys sanmingensis (Anura: Megophryidae)
Source: Mitochondrial DNA B Resour. 2026 Apr 29;11(6):712–6. doi: 10.1080/23802359.2026.2663604 (PMC13134743; doi:10.1080/23802359.2026.2663604)
Supplement: Supplementary material.pdf [file TMDN_A_2663604_SM4106.pdf]

Supplementary material

The complete mitochondrial genome of *Boulenophrys sanmingensis* (Anura: Megophryidae)

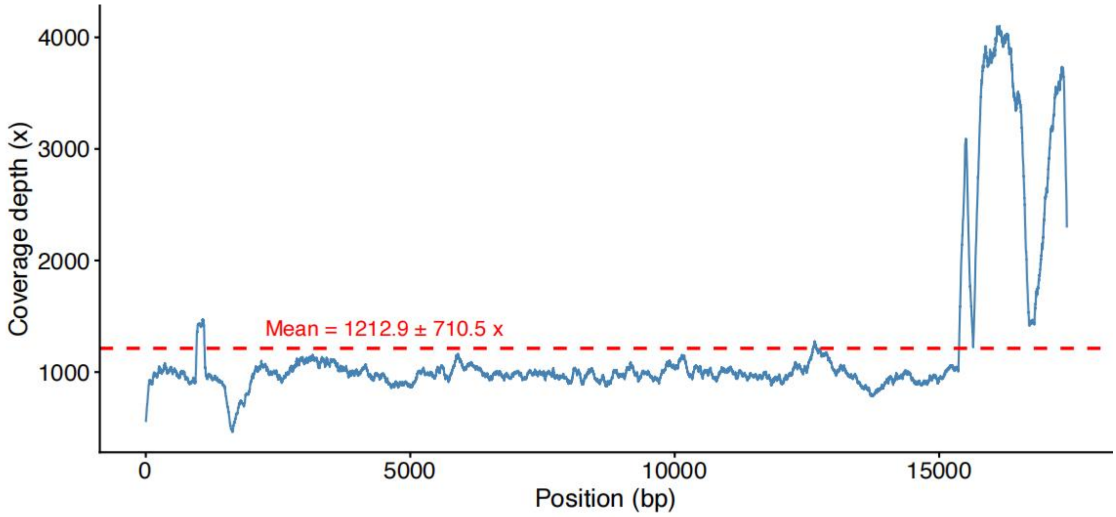

Figure S1. Coverage maps of the mitochondrial genomes for *Boulenophrys sanmingensis.*
